# Operationstechniken der Ober- und Unterschenkelamputation

**DOI:** 10.1007/s00113-024-01531-0

**Published:** 2025-01-31

**Authors:** Patrick Schröter, Marc Hückstädt, Steffen Langwald, Bianca Schröter, Philipp Kobbe

**Affiliations:** Klinik für Unfall- und Wiederherstellungschirurgie, BG Klinikum Bergmannstrost Halle, Merseburger Straße 165, 06112 Halle, Deutschland

## Einleitung

Amputationen oberhalb des Sprunggelenks, sog. Majoramputationen, wurden 2022 in Deutschland 16.384-mal bei insgesamt 67.331 Amputationen der unteren Extremität durchgeführt. Hauptursachen sind Gefäßerkrankungen und Diabetes mellitus. Seltene Ursachen (25–30 %) stellen Tumoren, Unfälle oder chronische Entzündungen wie Osteomyelitis dar. Nur etwa 30–50 % der Patienten erhalten im Rehabilitationsprozess Prothesen. Neben den Komorbiditäten stellt eine ungenügende Amputationstechnik einen wesentlichen Grund für nichtbelastbare Stümpfe da. Die meisten Amputationen sind planbar; funktionelle Bedürfnisse, Komorbiditäten und orthopädietechnische Versorgungsmöglichkeiten müssen im Vorfeld berücksichtigt werden.

## Vorüberlegungen

Das Ziel der Amputation ist ein gut belastbarer Stumpf. Dabei bestehen Anforderungen an die Stumpfbeschaffenheit. Bei Planung der Amputationstechnik sind folgende Stumpfeigenschaften anzustreben.

Haut- und Unterhaut:

- gute Durchblutung (Rekapillarisierungszeit ca. 2–3 s),
- keine Sensibilitätsstörung der Haut,
- schmerzfrei,
- Haut/Narbe ist über dem Untergrund verschieblich und reizlos,
- Lastaufnahme wird vertragen.

Weichteile:

- fixierte Muskeldeckung des Stumpfes unter physiologischer Vorspannung,
- große Gefäße sind kurz proximal des knöchernen Stumpfes chirurgisch abgesetzt,
- die Nerven (Oberschenkelstumpf: N. femoralis, N. ischiadicus; Unterschenkelstumpf: N. tibialis, Nn. fibulares, N. suralis) sind hinreichend gekürzt und in die Weichteile verlagert.

Knöcherner Stumpf:

- die knöcherne Resektionsfläche ist an den Kanten abgerundet,
- bei transtibialer Amputation ist die Vorderkante angeschrägt,
- es bestehen keine störenden Ossifikationen/Osteophyten,
- beim Burgess-Stumpf ist die Fibula ist ca. 1 cm kürzer als die Tibia und leicht ansteigend von medial nach lateral angeschrägt, die Membran zwischen Fibula und Tibia ist intakt,
- beim Ertl-Dederich-Stumpf: stabile Synostose zwischen Tibia und Fibula

## Oberschenkelamputation

Oberschenkelamputationen sind Amputationen durch die Femurdiaphyse. Der Stumpf wird mit einem myokutanen Weichteillappen bedeckt, um ein belastbares Polster zu schaffen. Die transkondyläre Amputation am Femur zählt aufgrund ihrer biomechanischen Eigenschaften zu den Knieexartikulationen und zeichnet sich durch eine fasziokutane Weichteildeckung ohne Muskelkomponente aus. Unter Berücksichtigung einer suffizienten Weichteildeckung ist stets der längstmögliche Stumpf zu wählen. Je kürzer der Femurstumpf, umso mehr überwiegen die Muskelkräfte um die Hüftbeugung und die Außenrotation. Es resultieren eine vermehrte Abduktions‑, Außenrotations- und Flexionsfehlstellung des Femurs. Gezielte muskelrefixierende Techniken (Myodese, Myoplastik, transossäre Myopexie, M.-adductor-magnus-Technik nach Gottschalk) bewirken eine physiologischere Stellung des Femurs.

### Merke.

Je länger der mechanische Hebelarm ist, desto günstiger sind die Übertragung von Kräften und der resultierende Energieaufwand beim Gehen.

### Indikation

- Nichtbeherrschbare Infektionen des Unterschenkels und Kniegelenks,
- keine Belastbarkeit bei chronischen Wunden,
- Verletzungen ohne Erhaltungsmöglichkeit der Extremität,
- Tumoren,
- schwere Perfusionsstörungen,
- selten kongenitale Fehlbildungen.

### Kontraindikation

- Tieferes Amputationsniveau (Bsp. Knieexartikulation).

### Operationsablauf

#### Lagerung

Der Eingriff erfolgt in Rückenlage. Die ipsilaterale Gesäßseite ist durch ein geeignetes Lagerungshilfsmittel anzuheben. Hierdurch wird die intraoperative Stumpfexposition verbessert. Auf der Operationsseite wird der Unterbauch steril eingedeckt, um bei Bedarf einen sicheren Zugang zur Gefäß-Nerven-Achse oder zu Gefäßprothesen zu gewährleisten. Eine sterile Blutsperre ist bei langen Oberschenkelstümpfen hilfreich, um den Blutverlust zu reduzieren.

#### Resektionsphase

**Abb. 1:**
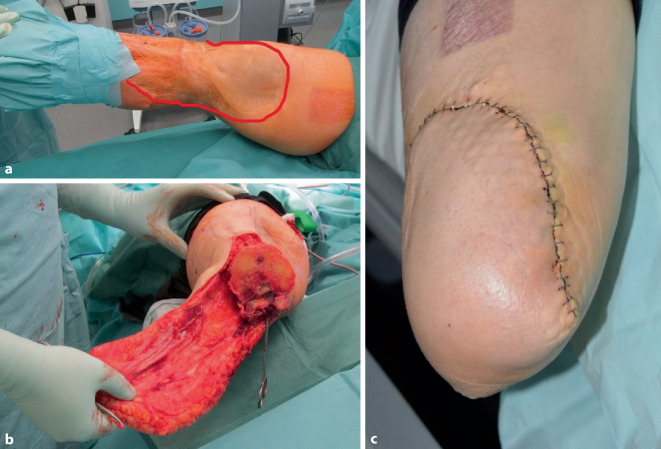
Atypische Hautlappenführungen sind u. U. notwendig. Große Spalthautareale am Stumpfende würden bei klassischem Fischmaulschnitt in diesem Fall zu einer schlechten Stumpfbelastbarkeit führen (**a**). Bei rezidivierenden Weichteilinfektionen und bestehender Kniearthrodese wurde ein langer dorsolateraler fasziokutaner Lappen (**b**) so gewählt, dass eine Vollhautdeckung des Stumpfes erreicht werden konnte (**c**)

Für die Hautschnittführung bestehen verschiedene Möglichkeiten:

- Vorderer und hinterer Hautlappen als Fischmaulverschluss: Quadrizepsmuskulatur bildet den vorderen, hintere Beugemuskulatur und Adduktorengruppe bilden den hinteren Muskellappen.
- Sagittale Schnittführung: mediale und laterale Hautlappenbildung mit analoger Muskelpräparation in der Tiefe.
- Atypische Hautlappen können für den Weichteilverschluss zur Stumpfdeckung genutzt werden (Abb. 1).

Grundsätzlich sollte die Hautnaht nicht unmittelbar am Stumpfende zu liegen kommen, um beim Tragen des Prothesenschaftes auf die Narbe keine Distraktions-, sondern Kompressionskräfte auszuüben.

Haut- und Unterhautschnitt entlang vorgezeichneter Linien. Nach Durchtrennung der Muskelfaszie werden die relevanten Muskelgruppen identifiziert und etwa 4–6 cm distal der geplanten Knochenabsetzung mittels Elektrokauter oder scharfem Amputationsmesser durchtrennt. Durch diesen Muskelüberstand ist später eine effektive Muskeldeckung des Knochenendes durch Myodese oder Myoplastie möglich.

Die A. und V. femoris superficialis werden im Adduktorenkanal identifiziert und abgesetzt. Danach wird das Femur von ventral aufgesucht und das Periost inzidiert. Die Osteotomie erfolgt entweder mit einer oszillierenden Säge oder einer Gigli-Säge, stets unter Kühlung durch eine Spüllösung. Anschließend werden alle vorhandenen Knochenkanten geglättet.

Hiernach wird das Femur angehoben, und die dorsalen Weichteile werden präpariert. Dabei werden die A. und V. femoralis profunda dargestellt und ligiert.

Der N. ischiadicus im hinteren, der N. saphenus im medialen und der N. femoralis im vorderen Kompartiment werden identifiziert und ca. 5 cm proximal des knöchernen Stumpfendes zurückgekürzt und in muskuläre Weichteile verlagert, um die Entstehung schmerzhafter Stumpfneurome zu reduzieren. Aufgrund der größeren Vasa nervorum empfiehlt es sich, die Nervenstümpfe des N. ischiadicus und des N. femoralis zu ligieren.

#### Rekonstruktionsphase

Bei der klassischen Myoplastie werden die antagonisierenden Muskelgruppen über dem knöchernen Stumpfende miteinander vernäht. Bei der Myodese werden die präparierten Muskelgruppen zusätzlich durch Bohrlöcher am Knochen fixiert. Es werden 2-mm-Bohrlöcher knapp oberhalb des Femurendes an den vorderen, hinteren, medialen und lateralen Seiten unter Kühlung mit Spüllösung gesetzt. Die Muskulatur wird mit langsam resorbierbarem Nahtmaterial am Knochen unter Vorspannung fixiert. Das Femur wird dabei vollständig gestreckt und nach innen geführt.

**Abb. 2:**
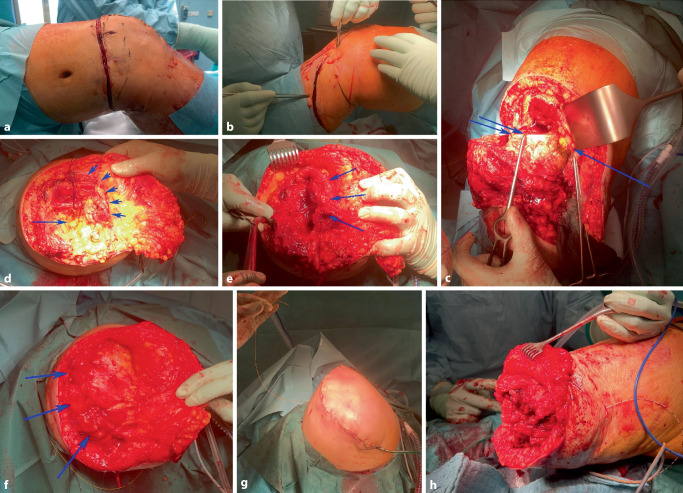
Aufgrund rezidivierender Infektionen im Kniegelenk wurde die Indikation zur Oberschenkelamputation gestellt. Bei Amputationen im distalen Drittel sollte eine Gottschalk-Plastik erfolgen. Hierbei wird medial (**b**) über den Adduktoren ein längerer Hautunterhautlappen entwickelt, und lateral erfolgt horizontale Schnittführung (**a**). Die Sehne des M. adductor magnus (**c**, *ein* *Pfeil*) wird vom Tuberculum adductorium gelöst und erhalten. Anschließend erfolgt die Osteotomie am metadiaphysären Übergang des Femurs (**c**, *2* *Pfeile*). Das Gefäß-Nerven-Bündel und der N. ischiadicus (**d**, *ein* *Pfeil*) werden über Ligaturen versorgt. Die Adduktoren und Teile des M. vastus medialis werden so lang wie möglich erhalten, über das Femurende geschlagen und mittels Myodese und Myoplastie lateral distal fixiert (**d**–**f**, *Pfeile*). **g** zeigt den Wundverschluss mit lateral gelegener Narbe und dem sichtbaren Schmerzkatheter, welcher am N. ischiadicus zu liegen kommt. Zum Vergleich zeigt (**h**) bei möglicher Vollhautdeckung des Stumpfendes einen klassischen Fischmaulschnitt mit Fixierung der antagonisierenden Muskeln am knöchernen Ende. Zudem ist hier der Schmerzkatheter des N. ischiadicus zu erkennen

Gottschalk beschrieb die Präparation des M. adductor magnus, dessen Sehne von medial über das distale Ende des Femurs zur lateralen Seite geführt und dort durch 2‑mm-Bohrlöcher mit langsam resorbierbarem Nahtmaterial fixiert wird. Dabei ist das Femur in maximaler Adduktion zu halten. Anschließend wird die Quadrizepsmuskulatur ebenfalls über das Femurende geführt und an dessen Rückseite mit der Beugemuskulatur und über Bohrlöcher vernäht. Die Hüfte ist dabei vollständig gestreckt (Abb. 2).

Der Wundverschluss erfolgt mehrschichtig: Zuerst wird die Faszie verschlossen, gefolgt von der subkutanen Gewebeschicht und schließlich der Haut. Die Hautnaht sollte spannungsfrei sein, um Hautnekrosen zu verhindern. Der sterile Wundverband mit durchblutungsangepasster elastokompressiver Wickelung wird angelegt.

## Unterschenkelamputation

Als Unterschenkelamputation werden die Bereiche zwischen der auf Höhe des Sprunggelenks durchgeführten Syme-Amputation und der proximalen Amputation mittig des Ansatzes der Patellarsehne an der Tuberositas tibiae bezeichnet. Die Syme-Amputation unterscheidet sich hinsichtlich der biomechanischen Eigenschaften und der späteren Prothesenversorgung erheblich von einer distalen Unterschenkelamputation und wird deshalb zu den endbelastbaren Rückfußamputation gerechnet.

Die Operationstechnik von Sir Ernest Burgess ist weltweit die häufigste Methode zur Unterschenkelamputation. Bei Patienten mit arterieller Verschlusskrankheit im Stadium IV nach Fontaine treten Wundheilungsstörungen in der Regel über der anterolateralen Muskulatur und Haut auf. Die Unterschenkelamputation nach Brückner stellt für dieses Patientenkollektiv eine wertvolle Modifikation der Burgess-Amputation mit Entfernung der anterolateralen Muskelgruppe und der Fibula dar. Es können Wundheilungsstörungen vermindert und somit das Kniegelenk häufiger erhalten werden.

Für Patienten mit traumatischer Amputationsursache bietet die Technik nach Ertl/Dederich eine Möglichkeit, einen längeren und endbelastbareren Stumpf zu rekonstruieren.

### Indikation

- Nichtbeherrschbare Infektionen des distalen Unterschenkels bzw. Rückfußes,
- keine Belastbarkeit bei chronischen Wunden,
- Verletzungen ohne Erhaltungsmöglichkeit der Extremität,
- Tumoren des distalen Unterschenkels oder Rückfußes,
- schwere distale Perfusionsstörungen,
- selten kongenitale Fehlbildungen.

### Kontraindikation

- Mögliche distale Syme-Amputation,
- Osteitis des Tibiakopfes,
- s, Indikation Brückner-Amputation

### Operationsablauf

#### Vorbereitung

Die Operation erfolgt in Spinalanästhesie oder in einer Kombination eines distalen Ischiadikusblocks/Femoraliskatheters unter Vollnarkose. Die permanente perioperative Nervenblockade nach zentralnervös vermindert das Risiko von Phantomschmerzen oder Neurombeschwerden. Der dorsale Hautlappen sollte anfänglich so lang wie möglich erhalten bleiben, um die Narbe später optimal in den vorderen Bereich des Unterschenkelstumpfes zu verlagern.

#### Lagerung

Der Patient liegt auf dem Rücken. Ein elektrische Beinhalter verbessert die Sicht und den Komfort während der Operation. Bei fehlenden Kontraindikationen erleichtert eine Blutsperre das Vorgehen im Rahmen der Resektionsphase. Der mögliche intraoperative Wechsel auf ein höheres Amputationsniveau, wie eine Knieexartikulation, ist bei der Lagerung zu berücksichtigen. Wichtige anatomische Landmarken und die geplante Schnittführung sollten eingezeichnet werden.

### Operationstechnik nach Burgess

#### Resektionsphase

Zunächst wird eine Inzision in Höhe an Kutis und Subkutis des ventralen Hautlappens durchgeführt. Als Orientierung empfiehlt es sich, den Übergang des Muskelbauches des M. gastrocnemius zur Achillessehne aufzusuchen. Anschließend erfolgt die Inzision ventral mittig zwischen diesem Punkt und der Tuberositas tibiae. Die Präparation erfolgt senkrecht bis zur Faszie des anterolateralen Kompartiments und der ventromedialen Tibia. Danach durchtrennt man die anterolaterale Muskulatur bis zur Membrana interossea, wobei das Gefäß-Nerven-Bündel um die A. tibialis anterior und den tiefen Ast des N. fibularis freigelegt und über Ligaturen durchtrennt wird. Die medial subkutan befindliche V. saphena magna wird ebenfalls ligiert. Ein identifizierbarer N. saphenus ist nach proximal, aus dem zukünftigen Narbenbereich heraus, zu kürzen. Nun wird das Periost der Tibia und Fibula mit einem Skalpell inzidiert und mit einem Raspatorium nach distal abgeschoben. Dabei ist das proximale Periost zu schonen.

Jetzt wird das Amputationsmesser unmittelbar hinter der Fibula bis zur dorsomedialen Tibiakante durchgestoßen und unter Bildung eines dorsalen Muskelhautlappens hinter den Knochen weit nach distal geführt. An der distalen Grenze des Lappens wird das Messer senkrecht gedreht, um die Weichteile zu trennen. Diese Methode ermöglicht es, alle Weichteile bis auf wenige Fasern des M. tibialis posterior effizient zu lösen.

Es ist nun ratsam, zuerst die Fibula zu osteotomieren, um Splitterbrüche durch die Instabilität des Unterschenkels zu vermeiden. Die Fibula wird etwa 1 cm oberhalb der geplanten Tibiaresektionshöhe freigelegt und unter leichtem lateralen Anschrägen osteotomiert. Danach wird die Tibia in anterior-posteriorer Richtung durchgesägt. Es ist stets ausreichend durch Spülung zu kühlen, um Nekrosen am Resektionsrand zu verhindern. Die ventrale Tibiakante wird angeschrägt, um ventrales Anschlagen bei der Prothesennutzung zu vermeiden. Danach werden die Kanten der Tibia und Fibula mit einer Knochenfeile abgerundet.

Nun müssen noch unversorgte großen Unterschenkelgefäße identifiziert und ligiert werden. Es folgt das Öffnen einer möglichen Blutsperre. Anschließend sind die vorhandenen Weichteile gründlich zu inspizieren. Findet sich eine bisher nicht erkannte Weichteil- oder Knocheninfektion bzw. eine unerwartet starke Durchblutungsstörung, ist evtl. eine Änderung der Amputationstechnik nötig. Bei höhergradiger Durchblutungsstörung wird auf die Brückner-Amputation gewechselt. Sind nach Resektion von infiziertem Gewebe keine ausreichenden Weichteile mehr zur Deckung des Stumpfs vorhanden, so muss weiter proximal im Sinne einer Knieexartikulation amputiert werden. Ein dorsaler Fasziokutanlappen ist zum Decken der Femurkondylen sehr gut geeignet.

Der M. soleus ist zu entfernen. Beim nichtvoroperierten Patienten kann man sehr gut am medialen Rand des M. soleus mit dem Finger zwischen diesen und dem M. gastrocnemius medialis fahren und diese beiden Muskeln stumpf voneinander lösen. Die Sehne des M. plantaris, wenn sie vorhanden ist, kann zwischen beiden Muskeln gefunden werden und, dient dann als anatomische Landmarke. Lateral muss die Peronäalmuskulatur scharf durchtrennt werden. Anschließend kann wieder stumpf mit dem Finger unter sanften Zug nach kaudal der M. soleus vom M. gastrocnemius vollständig separiert werden. Der M. soleus wird nach proximal dorsal der Tibia verfolgt und von der hinteren Fläche der Tibia nahezu vollständig entfernt. Seine Ansatzfasern an der Rückfläche der Fibula werden belassen. Das nahezu vollständige Entfernen des M. soleus, welcher bei Belassen zu Venenthrombosen und Nekrosen neigt, hat zudem den Vorteil, dass das Gefäß-Nerven-Bündel um die A. tibialis posterior sehr gut einsehbar ist und sicher proximal des knöchernen Resektionsrands versorgt werden kann. Es kann durch diese deutliche Volumenreduktion der Muskelbauch des Gastroknemius medialis sicher über die Tibia geschlagen werden. In die entstandene Wundhöhle an der Tibiarückfläche sollte eine Redon-Drainage eingelegt werden.

**Abb. 3:**
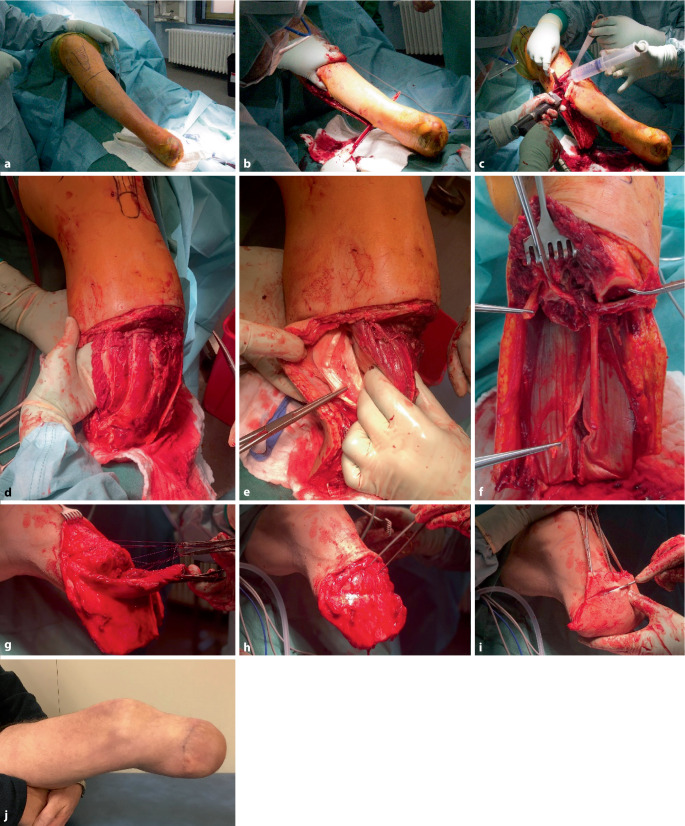
Darstellung des Vorgehens der Unterschenkelamputation nach Burgess. Lagerung auf dem elektrischen Beinhalter, angelegte Blutsperre und präoperatives Anlegen eines distalen Ischiadicusblock sowie proximalen Femoraliskatheter (**a**). Wenn eine Blutsperre möglich ist erfolgt das scharfe Durchtrennen der anterolateralen Muskelgruppe und das Bilden eines myofasziokutanen Lappens mit dem Amputationsmesser (**b**). Hiernach erfolgen die Osteotomien der Fibula und anschließend der Tibia (**c**). Von medial kann in der Regel stumpf zwischen dem Musculus gastrocnemius medialis und dem Musculus suralis eingegangen werden. Als Leitstruktur kann hier die Sehne des Musculus plantares dienen (**d**, **e**). Nach Entfernung des Musculus suralis und der Zehenbeuger werden die Gefäß- und Nervenstrukturen aufgesucht. Die 4 Hauptunterschenkelnerven sollten gefunden und gekürzt werden (**f**). Um eine bessere Muskelfixierung am Knochen zu erreichen führen wir eine tiefe (**g**) und eine oberflächliche Myoplastie (**h**) durch. Bei geringem prätibialen Weichteillager ist unter Umständen auch eine Myodese notwendig. Entscheidend für das langfristig günstige Ergebnis ist ein Überschlagen der Muskulatur über den knöchernen Stumpf und Fixieren unter physiologischer Vorspannung. Zum Hautverschluss werden die ventralen Haut- und Unterhautweichteile so ein gekürzt, dass die resultierende Narbe prätibial des Stumpfendes zu liegen kommt (**i**). Ausheilungsergebnis vor Beginn der Interimsprothesenversorgung (**j**)

Die Unterschenkelnerven werden aufgesucht und proximal in den Weichteilen über Ligaturen abgesetzt. Der N. tibialis wird ca. 3–4 cm proximal der Tibiaresektion eingekürzt. Der N. peronaeus teilt sich häufig proximal des Fibulaköpfchens in den oberflächlichen und tiefen Ast. Beide Nervenäste müssen etwa 2–3 cm proximal des Fibularesektionsrands durchtrennt und in die Muskelweichteile verlagert werden. Der N. suralis lässt sich sehr gut zwischen den beiden Muskelbäuchen des Gastroknemius finden und ist ebenfalls zu kürzen (Abb. 3).

#### Rekonstruktionsphase

Nachdem die Resektionsphase der Operation abgeschlossen ist, gilt es, eine gute Weichteildeckung des Stumpfes sicherzustellen. Aus diesem Grund sollte initial der dorsale Lappen sehr lang gewählt sein. Die Muskelbäuche der Mm. gastrocnemius medialis und lateralis werden nun ohne Separation vom Unterhautfettgewebe über die Tibiaresektionsfläche gelegt. Findet sich ein gutes Nahtlager am Periost und an der ventralen Faszie, kann die Muskulatur durch resorbierbares Nahtmaterial fixiert werden. Reicht dieses Nahtlager nicht aus, ist eine transossäre Fixierung als Myopexie nötig.

Insbesondere bei älteren Patienten kann es leicht zum Reißen der Muskelnähte kommen. Deswegen sollte als Nahtlager die am Muskel belassene oberflächliche Unterschenkelfaszie zur Naht mitgenutzt werden. Die von dorsal herumgeschlagene Muskulatur sollte mindestens einen, eher zwei Daumen breit ventral und proximal der tibialen Resektionsfläche zu liegen kommen, um eine sichere Muskelfixierung zu erreichen. Dies führt in den ersten Wochen zu einer leichten ventral gelegenen semizirkulären Weichteilwulst. Diese bildet sich im Rahmen der Stumpfkonditionierung jedoch vollständig zurück.

Nach der Muskeldeckung erfolgt das Kürzen der Kutis und Subkutis. Bei guter dorsaler Lappendurchblutung ist der ventrale Hautlappen so einzukürzen, dass ein spannungsfreier Wundverschluss mit Narbenposition proximal der Muskelfixierung möglich ist. In den Subkutanraum kann eine Redon-Drainage eingelegt werden. Die Subkutannähte sind tiefgreifend und kutisnah anzulegen, damit eine möglichst vollständige Hautadaptation erreicht wird. Einzelknopfnähte der Haut sind zu bevorzugen, um die Durchblutung nicht zu kompromittieren. Bei ausgeprägten Durchblutungsstörungen kann zum Vermeiden von Hautnekrosen durch Einzelknöpfnähte der Wundverschluss durch breite Steri-Strips erfolgen.

Eingebrachte Redon-Drainagen, insofern sie weit proximal ausgeleitet wurden, müssen nicht zwingend angenäht werden. Dies gestattet bereits am ersten postoperativen Tag ein Lupfen der Drainagen, um evtl. Blutkoagel von den Drainagelöchern zu lösen. Der Wundverband erfolgt mit sterilem Pflaster und einer lockeren elastokompressiven Wickelung bis zur Mitte des Oberschenkels.

## Operationstechnik nach Brückner

### Indikation

- Siehe Burgess-Amputation, aber zusätzlich:
- AVK-Stadium 4,
- zerrissene Membrana interossea,
- kurze Stumpflänge mit Verlust der Membrana interossea.

### Kontraindikation

- Mögliche Burgess-Amputation,
- mögliche distale Syme-Amputation.

#### Resektionsphase

Die Operation beginnt ähnlich wie die Resektionsphase nach der Burgess-Technik. Die Tibiaosteotomie erfolgt jedoch etwa 10–12 cm unterhalb des Kniegelenks. Nach Entfernung des M. soleus und der Gefäß- und Nervenstrukturen wird das anterolaterale Muskelkompartiment reseziert. Die Faszie über dem M. tibialis anterior wird mit einem Abstand von 1 cm zur Schienbeinvorderkante längs gespalten, um den Muskel freizulegen und diesen zu entfernen. Die laterale Tibiafläche sollte nur noch von Periost bedeckt sein. Die A. tibialis anterior und ihre Begleitvenen werden proximal der Membrana interossea ligiert. Anschließend werden die Peronäalmuskulatur entfernt und die Fibula am proximalen Tibiofibulargelenk exartikuliert. In seltenen Fällen muss der prominente tibiale Anteil des Gelenks geglättet werden. Der N. peronaeus communis wird weit proximal abgesetzt, um Druck auf das Nervenende in der Prothese zu vermeiden.

**Abb. 4:**
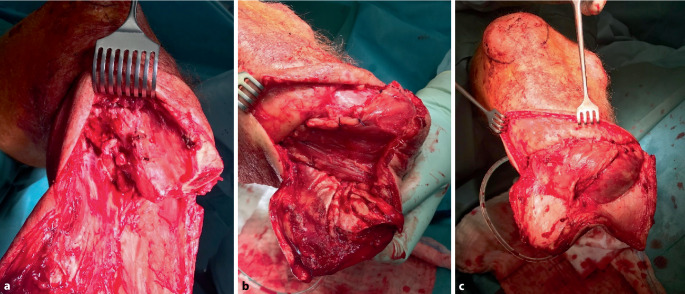
Das operative Vorgehen ist anfänglich der Burgess-Amputation analog. Aufgrund der schlechten Perfusion bei 4°pAVK erfolgt aber zusätzlich das vollständige Entfernen der anterolateralen Muskulatur und der Fibula (**a**). Bei der Stumpfrekonstruktion wird zunächst der M. gastrocnemius medialis über die Tibia in das ehemalige Lager des M. tibialis anterior geschlagen (**b**). Um Strecke gewinnen zu können, muss die Tibia im Vergleich zum Burgess-Stumpf i. d. R. einige Zentimeter kürzer osteotomiert werden. Der M. gastrocnemius lateralis wird anschließend über den M. gastrocnemius medialis und das Tibiaende geschlagen. Beide Muskeln umhüllen unter leichter Vorspannung das knöcherne Stumpfende

Nach Entfernung der zehenbeugenden Muskulatur von der Tibiarückfläche verbleiben nur die beiden Muskelbäuche der Gastroknemien. Der N. tibialis ist ebenfalls weit proximal zu resezieren. Zusätzlich ist der N. suralis zwischen den Gastroknemiusmuskelbäuchen aufzusuchen und proximal abzusetzen (Abb. 4).

#### Rekonstruktionsphase

Der M. gastrocnemius medialis wird über die Tibiastumpfspitze und den Tibiavorderrand in das ehemalige Bett des M. tibialis anterior unter leichter Vorspannung gelegt und mit resorbierbarem Nahtmaterial fixiert. Die belassene Faszienlefze dient als Nahtlager. Der M. gastrocnemius lateralis wird ebenfalls unter Vorspannung über den M. gastrocnemius mediales und die Tibia gelegt und sowohl am Periost als auch an den Faszien fixiert. Eine Redon-Drainage ist i. d. R. nur im Subkutanraum notwendig. Der subkutane Wundverschluss erfolgt mit resorbierbarem Nahtmaterial zur vollständigen Wundrandadaptation. Es sollten wenige Einzelknopfnähte zum Hautverschluss verwendet werden, um Mikroperfusionsstörungen zu vermeiden. Bei Rückstichtechnik nach Donati oder Allgöwer treten häufiger trianguläre Wundrandnekrosen in Richtung der Narbe ausgehend vom Stichkanal auf.

## Operationstechnik nach Ertl-Dederich

Diese Operationstechnik ermöglicht Unterschenkelamputationen im mittleren Drittel. Aufgrund der notwendigen Synostose und sicheren Weichteildeckung ist sie nur für Patienten ohne relevante Komorbiditäten geeignet, meist bei traumatischen Amputationsverletzungen. Bei tibiofibularer Instabilität oder einer erforderlichen Tibiakürzung auf unter 12 cm zur Bildung eines Periostschlauchs sollte eine modifizierte Brückner-Amputation durchgeführt werden. Die anterolaterale Muskulatur bleibt erhalten. Beim Kürzen der Nn. fibulares superficiales et profundus muss darauf geachtet werden, die Nervenäste zur ortsständigen Muskulatur nicht zu resezieren, um eine Denervationsatrophie zu vermeiden.

### Merke.

Die Synostose zwischen Tibia und Fibula am Stumpfende erhöht die Endbelastbarkeit, sorgt für ein konstanteres Stumpfvolumen und bietet eine größere lasttragfähige Stumpfoberfläche durch den längeren Stumpf.

### Indikation

- Zerreißung der Membrana interossea mit Instabilität zwischen Tibia und Fibula,
- Amputation im mittleren Unterschenkeldrittel,
- meist traumatische Amputation.

### Kontraindikation

- Mögliche distale Syme-Amputation,
- arterielle Verschlusskrankheit,
- Diabetes mellitus mit sekundären Krankheitsfolgen.

### Operationsdurchführung

**Abb. 5:**
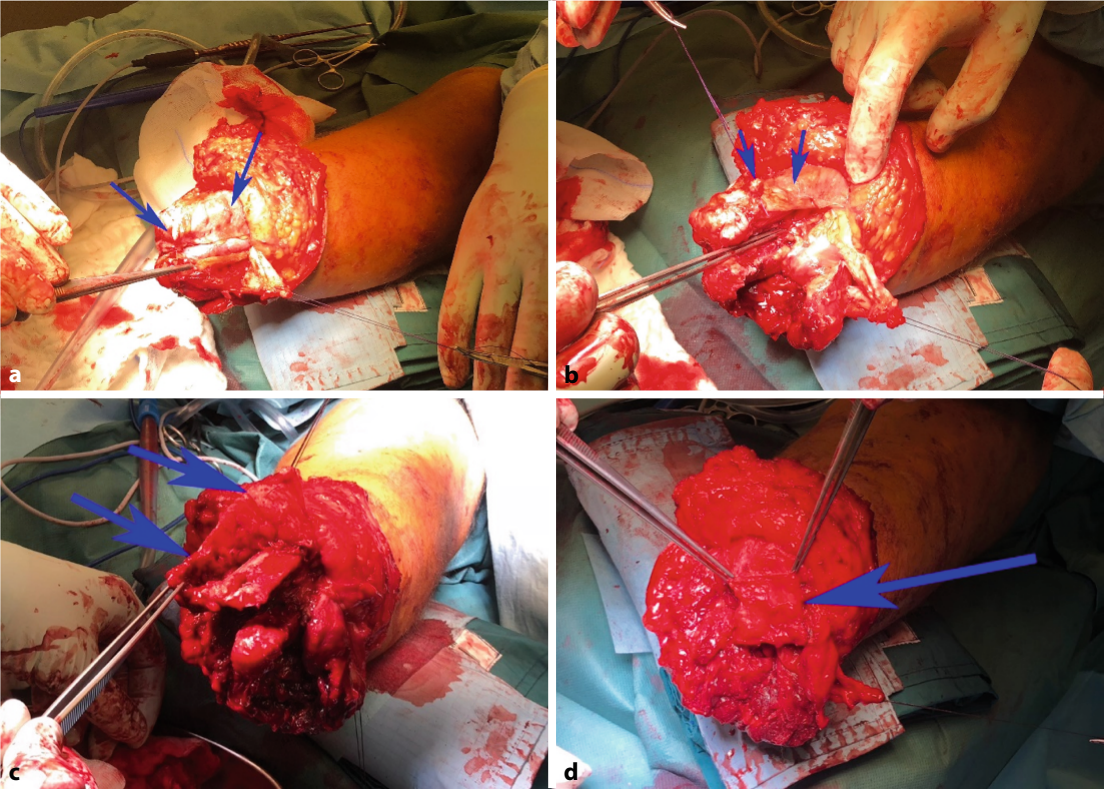
Bei der Ertl-Dederich-Rekonstruktion bei Unterschenkelamputation wird ein Periostschlauch mit „Kortikalis-Chips“ als Knochenbrücke zwischen Tibia und Fibula etabliert. **a**, **b** zeigen die Entwicklung eines Periostlappens (*Pfeile*) mit belassenen ca. 1–2 mm messenden Kortikalisanteilen, welche von der Tibia mit einem Meißel gelöst werden. In (**c**) ist der dicke Periostlappen mit der Kortikalis gut zu erkennen. Die Tibia wird um ca. 5 cm durch Osteotomie eingekürzt und der Periostschlauch zur Fibula gelegt (**d**, *Pfeil*). Dabei ist darauf zu achten, das Periost nicht zu verletzen und keine Kortikalisanteile distal des Schlauches in den Weichteilen zu belassen. Innerhalb von 6 bis 12 Monaten bildet sich eine stabile, großflächige Knochenbrücke aus

Je nach Art der Operation (stumpfkorrigierend oder primär) werden zunächst die distale Tibia und Fibula freigelegt. Die Instabilität zwischen Fibula und Tibia wird dabei deutlich sichtbar. Nun wird der Abstand zwischen der ventromedialen Tibia und der lateralen Fibula mit einem Lineal gemessen. Es ergibt sich die Länge des kortikoperiostalen Schlauches, der spannungsfrei angelegt werden muss. Das Periost der Tibia wird ventral und dorsal inzidiert, und zwei kortikoperiostale Lappen werden durch sukzessives Abschlagen von Kortikalisfragmenten gebildet. Es ist wichtig, das Periost nicht zu verletzen und sicherzustellen, dass die kortikalen Fragmente am Periost haften bleiben. Nach diesem Schritt erscheint die Tibia wie eine entrindete Korkweide. Die Periostlappen sollten bis etwa 1 cm proximal der geplanten Tibiaresektion geführt werden. Nun werden die Tibia und Fibula auf gleicher Höhe reseziert, wobei erneut darauf geachtet wird, das Periost nicht zu verletzen. Die Knochenkanten werden abgerundet, insbesondere die mediale tibiale Kante, um den Periostlappen leichter über die Tibia legen zu können. Die Periostlappen werden locker mit resorbierbaren Nähten adaptiert und bilden so den Periostschlauch. Der mediale Periostlappen wird etwa 15 mm länger gewählt, über die Fibula gelegt und mit resorbierbarem Nahtmaterial am Fibulaperiost fixiert. Alle Kortikalisfragmente sollten sich innerhalb des Periostschlauchs befinden, um unerwünschte heterotope Ossifikationen zu vermeiden (Abb. 5).

Der Periostschlauch wird anschließend mit einem Weichteillappen bedeckt. Da es meist um mittlere oder längere Stümpfe geht, sollte der besser durchblutete Hautlappen für den Wundverschluss gewählt werden. Für die spätere prothetische Versorgung ist es unerheblich, ob die Narbe ventral oder dorsal liegt.

#### Merke.

Sobald im konventionellen Röntgen erste Ossifikationstendenzen des Periostschlauchs sichtbar sind, empfehlen wir den Beginn der Interimsprothesenversorgung. Es sollten jedoch mindestens 12 postoperative Wochen abgewartet werden.

## Nachbehandlung nach Amputationen

Die ersten Wochen nach einer Amputation sind entscheidend für den späteren Prothesengebrauch. Die frühpostoperative Betreuung beginnt im Krankenhaus und markiert den Beginn der Rehabilitation. Wichtige Aspekte sind psychologische Unterstützung, Schmerztherapie und Stumpfbehandlung. Der Patient muss auf zukünftige Lebensumstände vorbereitet werden, inklusive täglicher Stumpfpflege und Informationen über den Rehabilitationsverlauf. Zu diesem Zeitpunkt sollte entschieden werden, ob eine Rehabilitation zur Verbesserung der Allgemeinkonstitution notwendig ist.

### Merke.

Die Rehabilitation mit einer ersten Interimsprothese beginnt frühestens ab der 6. postoperativen Woche. Vorzeitige Prothesenversorgung und Stumpfbelastungen führen oft zu dauerhaft schlechten Stumpfverhältnissen.

### Ödem‑/Kompressionstherapie

Der Stumpf sollte für einige Tage durch leichtes Unterpolstern hochgelagert werden, um das Ödem leichter zurückzubilden. Bei verlängerter Rekapillarisierungszeit ist eine flache oder leichte Tieflagerung zur Unterstützung der arteriellen Durchblutung notwendig.

Eine durchblutungsangepasste, kompressive Stumpfwicklung wirkt dem postoperativen Ödem entgegen. Initial sind Langzugbinden zu verwenden, um Drucknekrosen durch unerwartetes Anschwellen des Stumpfes zu vermeiden. Nach Entfernung der Drainagen und beginnendem Ödemrückgang wird auf eine dreilagige Wickelung mit Kurzzugbinden gewechselt. Der Verband reicht bei Unterschenkelamputationen bis zur Mitte des Oberschenkels, bei Oberschenkelamputationen soweit proximal wie möglich. Die Binden sind in narbenkomprimierender Zugrichtung anzulegen.

Bei Patienten mit starken Durchblutungsstörungen erfolgt die Kompressionswickelung über einer Lage aus Wattebinden. In den ersten Tagen ist bei diesen Patienten eine mehrfache tägliche klinische Kontrolle zwingend notwendig, um kompressionsbedingte Komplikationen zu vermeiden.

#### Merke.

Ein Amputationsstumpf muss stets gewickelt werden. Ohne Stumpfwickelung ist eine geeignete Formgebung während der Abheilung für die spätere Prothesennutzung nicht möglich.

### Stumpfschmerzen/Phantomschmerzen/Spiegeltherapie

Es empfiehlt sich, prä- oder perioperativ perineurale Schmerzkatheter zu legen. Zusätzlich kann eine PCA-Pumpe nach WHO-Empfehlungen verwendet werden. Starkes frühpostoperatives Schmerzerleben kann Phantomschmerzen begünstigen und sollte vermieden werden.

Phantomschmerzen treten selten vor Ende der ersten postoperativen Woche auf und sind nicht mit einem schmerzlosen Phantomgefühl zu verwechseln. Früh auftretende Phantomschmerzen können auf einen Frühinfekt, der die Nervenstümpfe reizt, hinweisen und sollten zu weiterführender Infektionsdiagnostik führen.

Die Spiegeltherapie wurde ursprünglich zur Behandlung von Schlaganfallpatienten eingesetzt. Durch Projektion der gesunden Körperseite im Spiegel auf die erkrankte Seite greift sie in die Reorganisation der Großhirnrinde ein und kann bei einigen Patienten zur Schmerzlinderung beitragen.

Medikamente wie Pregabalin oder Gabapentin, kombiniert mit Amitriptylin, haben sich ebenfalls als schmerzlindernd erwiesen.

#### Merke.

Schmerzmodulatorische Maßnahmen sind ab dem ersten postoperativen Tag zu beginnen.

### Physiotherapie

Bereits am ersten postoperativen Tag beginnen physiotherapeutische Behandlungen zur Erhaltung der Allgemeinkonstitution. Dabei darf auch das ipsilaterale Hüftgelenk beübt werden. Der Patient sollte sich mit Unterarmgehstützen mobilisieren und das Transfertraining vom Bett in den Rollstuhl und die Nasszelle üben. Zudem sollte er frühzeitig in die stumpfkonditionierende Kompressionsbehandlung einbezogen werden.

Da die Muskulatur durch Myoplastie oder Myodese fixiert wurde, ist eine aktive Beübung des proximalen Gelenks gegen Widerstand nicht ratsam. Myodesen benötigen 8 bis 12 Wochen zur narbigen Heilung. Lastfreie Bewegungen sind gestattet.

Stumpfabhärtende Maßnahmen wie Narbenmassagen, stufenweise Lastaufnahme am Stumpfende, wechselwarmes Duschen und mechanische Stumpfabhärtung erfolgen erst nach abgeschlossener primärer Wundheilung. Diese Behandlungen sind für den ambulanten Bereich oder die stationäre Prothesenrehabilitation vorgesehen.

